# Effects of a cafeteria-based sustainable diet intervention on wellbeing at a large German hospital: a quasi-experimental study

**DOI:** 10.1186/s12889-025-22533-6

**Published:** 2025-06-02

**Authors:** Laura Harrison, Alina Herrmann, Claudia Quitmann, Gabriele Stieglbauer, Christin Zeitz, Ulrich Reininghaus, Anita Schick, Ina Danquah

**Affiliations:** 1 Heidelberg Institute of Global Health, Medical Faculty and University Hospital, Heidelberg University, Im Neuenheimer Feld 130.3, Heidelberg, 69120 Germany; 2 Institute of General Medicine, University Hospital Cologne, Medical Faculty Cologne University, Kerpener Straße 62, Cologne, 50937 Germany; 3 ifeu - Institut für Energie- und Umweltforschung Heidelberg gGmbH, Wilckensstr. 3, Heidelberg, 69120 Germany; 4 Department of Public Mental Health, Central Institute of Mental Health, Medical Faculty Mannheim, Heidelberg University, J 5, Mannheim, 68159 Germany; 5 Centre for Epidemiology and Public Health, Health Service and Population Research Department, Institute of Psychiatry, Psychology & Neuroscience, King’S College London, London, UK; 6 German Center for Mental Health (DZPG), Partner site Mannheim-Heidelberg-Ulm, Mannheim, 68159 Germany; 7 Hertz-Chair Innovation for Planetary Health, Center for Development Research (ZEF), University of Bonn, Genscherallee 3, Bonn, 53113 Germany

**Keywords:** Plant-based diets, Mental wellbeing, Health-related quality of life, WEMWBS, EAT-Lancet Planetary Health Diet, Vegan

## Abstract

**Purpose:**

Sustainable diets are vital to tackle climate change and promote health. They may also offer significant benefits for mental and physical wellbeing. Creating a sustainable food environment at the working place could therefore contribute to the wellbeing of employees. In this quasi-experiment, we investigated effects of providing vegan menus and nutritional information in a cafeteria of Heidelberg University Hospital on mental wellbeing and health-related quality of life (HRQoL) among customers.

**Methods:**

Regular customers (> one visit/week) in the largest cafeteria (intervention group, *n* = 121) and in all other cafeterias (control group, *n* = 128) completed a questionnaire before and after the 3-month intervention period (16/01/2023–06/04/2023). We measured mental wellbeing using the Warwick-Edinburgh Mental Wellbeing Scale (WEMWBS), HRQoL using the Short Form- 36 Questionnaire (SF-36) and adherence to the Planetary Health Diet (PHD) using a Food Frequency Questionnaire. Difference-in-differences (DID) were calculated for the intervention effects on WEMWBS and SF-36 scores. Cross-sectional associations of the PHD-Index with these scores were calculated at baseline.

**Results:**

In this study population (*N* = 249; women: 65%), the mean baseline WEMWBS was 54.46 (standard deviation, SD: 7.13) in the control group and 55.19 (SD: 6.41) in the intervention group. The difference-in-differences was 0.61 (95% confidence interval, CI: - 0.78, 1.99; *p* = 0.39). Effects were insignificant for all SF-36 outcomes. Adherence to the PHD-Index was positively associated with self-rated health (beta: 0.48; 95% CI: 0.20, 0.75; *p* <.001).

**Conclusion:**

This worksite cafeteria-based diet intervention yielded nominal improvements in mental and physical wellbeing among customers; this could be mediated by increased adherence to the PHD. These trends warrant verification in larger-sized intervention studies with more intense intervention dosages. Our findings underline the importance of sustainable food environments for planetary health.

The protocol was registered at the German-Clinical-Trial-Register on 22/04/2024 (DRKS00032620).

**Supplementary Information:**

The online version contains supplementary material available at 10.1186/s12889-025-22533-6.

## Copyright

*Warwick-Edinburgh Mental Wellbeing Scale (WEMWBS)* © University of Warwick, 2006, all rights reserved. S(WEMWBS) was developed by the Universities of Warwick, Edinburgh and Leeds in conjunction with NHS Health Scotland.

*Warwick-Edinburgh Mental Wellbeing Scale (WEMWBS) (German Version): *© NHS Health Scotland, University of Warwick and University of Edinburgh, 2007, all rights reserved. Deutsche Übersetzung entwickelt von Almut Bachinger und Gert Lang, Forschungsinstitut des Roten Kreuzes, 2013, alle Rechte vorbehalten. (German Translation developed by Almut Bachinger und Gert Lang, Research Institute of the Red Cross, 2013, all rights reserved).

## Background

The concept of planetary health acknowledges that the health of each individual is dependent on and inseparable from the health of the planet [1]. In the year 2024, this planet has already been pushed over six out of nine planetary boundaries [2]. Following the 2021 report by the Lancet Countdown, global climate change due to greenhouse gas emissions is the largest threat to health now and in future [3]. The food sector is responsible for approximately one third of these emissions [4]. Therefore, to stay within planetary boundaries and reach the goals of the Paris Agreement [5], “sustainable diets” are imperative to be implemented [6].

Sustainable diets are defined as “diets with low environmental impacts that contribute to food and nutrition security and to healthy lives for present and future generations.” [7]. Sustainable diets can include pescetarian, vegetarian and vegan diets [6]. The “Planetary Health Diet” (PHD) designed by the EAT-Lancet Commission is mostly plant-based and advocates for reduced intakes of poultry, fish, processed and red meats, added sugar, and refined grains. In general, reducing meat and dairy consumption is the most important step towards more sustainable diets [6]. In the latest representative German national food consumption survey, Germans consumed around 3-times the amount of meat, 2-times the amount of dairy and only one-third of the amount of vegetables and legumes recommended in the PHD [8]. Furthermore, only 2% of Germans in a 2023 representative survey described themselves as vegan and only 8% as vegetarian [9].

To kick-off the global shift towards more sustainable dietary patterns, strategies to promote behavior change are urgently needed [6]. Facility-based diet interventions which modify the food environment, have proven effective in promoting sustained long-term diet change, in contrast to individual-level interventions that often yield higher but only short-term effects [10, 11]. So far, there has been little research on the effects of sustainable diet interventions in a working place food environment on mental and physical wellbeing.

Sustainable diets have beneficial effects on the planet, provide more adequate nutrient intake than average dietary patterns and have multiple co-benefits for human health [6]. However, although the EAT-Lancet Commission reported inverse associations of foods in the PHD with physical illnesses such as diabetes, cancer, and cardiovascular diseases [12], it did not report evidence on effects of this diet on mental and physical wellbeing. Previous research provides evidence for a beneficial association between the healthy diets and mental wellbeing [13]. However, further research on the relationship with sustainable diets is needed.

So far, cross-sectional studies, prospective cohort studies, and their meta-analyses have consistently reported associations of high fruit and vegetable intake [14, 15], the Mediterranean diet [16–18] and Mediterranean-DASH Intervention for Neurodegenerative Delay (MIND) diet [19] with improved mental health and wellbeing and slower cognitive decline [20]. Randomized controlled trials examining the Mediterranean diet or high fruit- and vegetable intake have shown that individuals in the experimental group reported improved mental wellbeing and reduced depression indices [21, 22]. Specific nutrients found in these foods and diets, including B vitamins and magnesium [23], have direct effects on multiple biological processes related to mental health [21, 22]. Indeed, recommendations of the above-mentioned diets overlap with those in the Planetary Health Diet, including high intakes of fibre, fruits and vegetables, isoflavonoids, vitamins, omega- 3-fatty-acids, and low intakes of saturated fats (e.g., from red and processed meats). Therefore, sustainable diets may have a positive effect on mental health. A recent study found that higher adherence to the EAT-Lancet diet was associated with lower risks of incident depression (hazard ratio: 0.81), anxiety (0.82), and their co-occurrence (0.76) [13].

Mental health can be defined as a “complete state of subjective well-being (i.e., hedonic and eudaimonic […]) as well as the absence of common mental disorders”. Hedonic wellbeing is the perception of pleasure and happiness in life. Eudaimonic wellbeing refers to living a meaningful life and striving to reach one’s full potential [24, 25]. Mental wellbeing can be seen as a continuum and thus, be described on a scale. Strong links have also been found between physical and mental health [26]. Given that individuals with lower levels of positive wellbeing are more likely to develop mental illness [27], it appears logical to centre mental health promotion on mental wellbeing. Interventions on the community- or institution-level can involve reducing risk factors and strengthening protective elements of mental health in an entire population [28–30].

Up to date, there are no studies on facility-based diet interventions to modify mental wellbeing, which rely solely on creating a more sustainable food environment in the working place. Furthermore, the association between the PHD and mental wellbeing has not yet been established. On this basis, our study aimed to determine the effects of sustainable menu options vs. the standard menu options in a cafeteria at a large university hospital on self-reported mental wellbeing and health-related quality of life (HRQoL) among employees. Furthermore, we aimed to define the relationships of the PHD with mental wellbeing and HRQoL.

## Methods

### Study design

This study was carried out in one of Germany's largest hospitals, Heidelberg University Hospital, featuring seven cafeterias for employees and guests. We conducted a quasi-experimental pre- and post-study with an intervention and control group. This study was part of a project, aiming to decrease greenhouse gas emissions from hospital supply chains [31].

### Intervention

Details of the intervention and the study design were previously published [32]. In brief, the intervention was provided for 3 months from 16/01/2023 to 06/04/2023. The standard lunch menu consisted of two meat-and-fish menu lines (75% of orders) and one vegetarian menu line (25% of orders in the previous year). The intervention program was designed by multiple stakeholders from hospital food supply, administration, and research. The intervention included two components: (1) one meat-and-fish-menu line was substituted with a vegan menu line (see Supplementary Fig. 1), and (2) information material on sustainable diets, their implications for health, and vegan online-recipes were distributed in the intervention cafeteria and per e-mail (see Supplementary Fig. 2).

### Intervention allocation

The intervention was allocated on a facility level. One hospital cafeteria (= intervention cafeteria) received the intervention. In all other cafeterias, the standard hospital meals remained unchanged (= control cafeterias). For this lifestyle intervention, allocation concealment and blinding were not possible. All cafeterias except two are on the university campus and are in a 8–20 min walking distance from the intervention cafeteria. The other cafeterias are in downtown (2 km) or in another area of the city (7 km).

### Participants

Hospital staff and other regular consumers of cafeteria meals, who were at least 18 years of age were eligible for participation. Participants who followed a medically prescribed dietary regimen were not eligible for participation. We recruited participants in front of the hospital cafeterias and through mailing lists. Participants who consumed cafeteria meals less than once a week during the intervention period were excluded from the analysis for the intervention effects on wellbeing outcomes. However, they were included in the cross-sectional analysis for the associations between the PHD-Index and wellbeing outcomes. The hospital cafeterias are mainly frequented by hospital employees, students of health sciences and volunteers, as well as few patient visitors. As patient visitors are unlikely to have visited the cafeterias on a regular basis over a duration of at least 4 months, we assume that the participants of our study had a working relationship with the hospital.

### Data collection

Through questionnaires, we collected demographic, socio-economic, lifestyle, dietary, and wellbeing data before and after the 3-month intervention period (baseline recruitment: 07/12/2022–15/01/2023, follow-up questioning: 27/03 − 30/04/2023). The questionnaires were distributed online and as paper-and-pencil version. Socio-demographic data comprised age, gender identity, marital status, income category, highest educational degree, number and age of children in the household, and occupation. Lifestyle data included the amount of moderate-intensity physical activity, and self-assigned type of dietary practice (vegan, vegetarian, pescatarian, flexitarian [meat/fish < three times/week], mixed diet). We assessed dietary intake of the last 3 months using a semi-quantitative food frequency questionnaire (FFQ) with 116 food items. We designed the FFQ explicitly for this study, aligning with the German food-based dietary guidelines [33] to encompass a wide range of plant-based food items. The assessments of mental and physical wellbeing are described below.

Intervention fidelity was measured at follow-up using three questions regarding (1) weekly frequency of cafeteria visits, (2) information material uptake, and (3) vegan meal consumption only in the intervention group.

### Outcomes

#### Primary outcome

The primary outcome of this study was mental wellbeing, measured by the Warwick-Edinburgh Mental Wellbeing Scale (WEMWBS) [34]. The WEMWBS is a 14-item questionnaire relating to an individual’s state of mental wellbeing (thoughts and feelings) in the previous 2 weeks [35]. Therefore, individuals who participated in the last two weeks of the intervention up to three weeks after the intervention were describing their wellbeing during the last month of the intervention up to the two weeks after. The WEMWBS items cover the following aspects of mental wellbeing: hedonic and eudemonic attributes like meaning, relaxation, social interest, vitality, self-actualization and problem-management, self-confidence, pleasure, interests and feeling loved. Responses range from 1 = “none of the time” to 5 = “all of the time” and are summed up to a minimum score of 14 and maximum score of 70. In a UK validation study, the WEMWBS showed good content validity and high internal consistency (Cronbach’s α = 0.91). In our sample, the WEMWBS also showed excellent internal consistency (Cronbach’s α = 0.90). A reliability study has shown that the retest reliability within one week is high (ICC = 0.83) and that the WEMWBS is responsive for group level analysis [36].

#### Secondary outcomes

Mental and physical wellbeing are strongly interconnected [26]. HRQoL is a construct that covers multiple dimensions of health, including physical wellbeing. Therefore, we administered additional items from the Short Form Health 36 (SF-36) questionnaire covering the following wellbeing outcomes [37, 38]: one question on global self-rated health and questions belonging to the four domains physical functioning, bodily pain, role limitations due to physical health (Role-Physical), and role limitations due to emotional problems (Role-Emotional). Each SF-36 item results in a discrete score between 0 to 100, with higher scores indicating better wellbeing. The global-self-rated health question is one question. It is a valid measure for subjective physical health but not for mental health [39, 40]. The four SF-36 scales (Physical Functioning and Bodily Pain, Role-Physical, and Role-Emotional) were constructed using the mean of the corresponding questionnaire items according to RAND scoring instructions [37]. In our sample, the Physical Functioning subscale consisted of 10 items (Cronbach’s α = 0.85), the Bodily Pain subscale consisted of 2 items (Cronbach’s α = 0.84), the Role-Emotional subscale consisted of 3 items (Cronbach’s α = 0.71) and the Role-Physical subscale consisted of 4 items (Cronbach’s α = 0.80). The SF-36 has been subject to many validity trials and the Physical Functioning subscale of the SF-36 was the best all-around measure of physical health until the year 2000 [41]. The reliability of these scales ranges from 0.82 to 0.93, making them a suitable measurement method for within-group comparisons [41]. Previous intervention studies have also found significant within-group-changes over time [42].

Lastly, we composed a PHD-Index according to Stubbendorff et al. using the FFQ dietary data. This index measures adherence to the PHD on a score from 0 (= least sustainable diet) to 42 score points (= most sustainable diet) [12]. For more frequent consumption, recommended food groups (*n* = 7) receive higher and unrecommended food groups (*n* = 7) receive lower score points [12]. For full details on the composition of the PHD-Index, see Supplementary Table 1.

### Sample size

We determined the required sample size for our study for our primary outcome mental wellbeing, assessed by the WEMWBS. Based on previous research on the effects of dietary changes on the WEMWBS, we calculated the sample size for paired t-tests (baseline and post-intervention) [15, 43–45]. We estimated the effect size to range between 0.18 and 0.30 WEMWBS points, at a significance level (α) of 0.05 and a power (1-β) of 0.8 [46]. This analysis indicated that we required a sample size ranging from 90 to 245 participants. To allow for 50% potential drop-outs, we aimed to enrol 500 individuals.

### Data analysis

All data analyses were conducted using SAS 9.4, with a significance threshold of α = 0.05 applied unless stated otherwise.

### Missing data handling

**Fig. 1:**
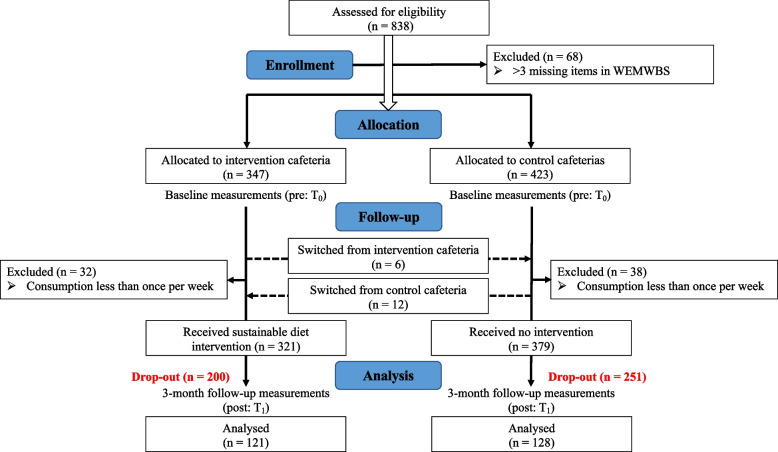
Flow diagram for quasi-experimental study

If participants had up to three missing items in the WEMWBS, these were imputed with the individual mean WEMWBS [35]. Participants with more than three missing WEMWBS items did not receive a score and were excluded from the analysis. If participants had any missing items in an SF-36 domain, this domain was coded as missing [37]. Figure 1 shows the flow chart for the quasi-experimental study, including 249 participants.

Regarding the construction of the PHD-Index we aimed to perform a complete-case analysis. If participants had only one missing item in the FFQ this missing item was rated as an inadvertent mistake and imputed with the sample mean. For participants with more than one missing item, the PHD-Index was coded as missing. This allowed the inclusion of an additional 49 participants.

If participants had missing SF-36 domains they were excluded from the analyses of these domains but not from this subsample (global self-rated health: *n* = 6, physical functioning: *n* = 9, bodily pain: *n* = 6, role-Physical: *n* = 9, role-Emotional: *n* = 10). We examined the residual plots of our outcomes and performed F-Tests and Levene’s Tests to check regression assumptions. To ensure homoscedasticity in our WEMWBS analyses, we removed seven outliers that exceeded 1.5 times the interquartile range of the pre-post differences. The cross-sectional distributions of the SF-36 outcomes are all left-skewed. Due to known ceiling effects in these outcomes [47], the mode of all SF-36 outcomes except for global-self rated health are at or close to 100. However, when examining the regression for the pre-post in-person differences SF-36 outcomes, there are only slight deviations in the histogram of the residuals and Q-Q plot. These deviations occur because the SF-36 outcomes are not continuous but discrete. However, for a large sample like ours (n > 200) the violation of the normal distribution is often regarded as uncritical, as the estimates of the regression coefficients are nevertheless reliable due to the central limit theorem. Furthermore, a previous study has used the same methods to describe effects on SF- 36 outcomes [42]. Individuals with extreme changes in SF-36 outcomes were excluded from the analysis, defined as changes > 70 percent points (global self-rated health: *n* = 1, physical functioning: *n* = 1, bodily pain: *n* = 0, role-physical: *n* = 17, role-emotional: *n* = 12). The number of individuals in the final datasets differs according to outcome and is annotated in the results tables. We found no significant effects in WEMWBS or SF-36 outcomes before removing outliers.

For the cross-sectional analysis for the associations between the PHD-Index and wellbeing outcomes, we created two subsamples of participants who had plausible FFQ data. One of these subsamples included the data from individuals with baseline examinations (*N* = 563), while the other dataset included data from participants with baseline and follow-up examinations (*N* = 182). Participants'dietary intakes were standardised to 2500 kcal/day before PHD-Index calculation. Participants with implausible energy intake (< 800 kcal/day or > 3500 kcal/day) were excluded from the analysis (*n* = 59). Participants with insufficient data to construct either the WEMWBS score or one of the SF-36 domains were excluded from this subsample (*n* = 13). See the flow diagram in Supplementary Fig. 3.

Participants with missing covariates were not excluded from the samples. In all adjusted models (Model 2 of the intervention sample and adjusted model of the PHD sample) and tables related to intervention feasibility, participants with missing covariate data were excluded only from the respective model or table. In the adjusted models, the individual who stated “diverse” gender was coded as missing. For the propensity-score-weighted model (Model 3 of the intervention sample), missing covariate values were imputed using the sample mode, with “diverse” participants also imputed as the sample mode (female).

### Descriptive statistics

For the quasi-experimental study, we compared demographic, socioeconomic, and lifestyle characteristics between the intervention and control group at baseline and follow-up, using Chi-square tests. Additionally, we calculated standardized mean differences of the baseline characteristics between groups. Lastly, we examined whether covariates with significant differences in groups or over time qualified as potential confounders and calculated their time-varying effects on the outcomes in the control group [48].

### Difference-in-differences analysis

We calculated the intervention effects using difference-in differences analyses and an as-treated analysis. An as-treated analysis examines participants in a study based on the treatment they actually received, regardless of the group they were originally assigned to [49]. This approach focuses on understanding the effects of the treatment as it was applied in real-world practice. We applied this method due to the possibility of switching groups in this study. In our as-treated analysis, participants were assigned to groups depending on the cafeteria where they most frequently consumed meals during the intervention period (specified at post-intervention).

The difference-in-differences analysis estimates the difference in the trend of an outcome between two or more time points in an intervention group and in a control group. It is based on the assumption that the outcomes of both groups would follow “parallel trends”, if there was no intervention [50].

In unadjusted linear regression analyses, we calculated mean values and standard deviations for all wellbeing outcomes for both groups and both time points, the effect estimates (difference-in-differences), their 95% confidence intervals (CIs), and p-values.

#### Sensitivity analyses

We investigated whether different dietary patterns at baseline (mixed, flexitarian, pescatarian, vegan, vegetarian) or the week in which participants completed the endline assessment influenced changes in mental well-being outcomes in both groups. For this we used multiple regressions. Also, to check the assumption that the difference-in-differences estimate remains stable when adjusting for differences in baseline characteristics, we performed a regression model adjusted for all available baseline covariates (Model 2) and a propensity score weighted model (Model 3). This propensity score describes the probability of being assigned to the intervention group or the control group conditioned on the same covariates as in Model 2. Propensity scores can serve as an alternative to covariate adjustment and are frequently used in difference-in-differences analyses to minimize baseline differences [51, 52]. It was possible for participants to switch between groups. Therefore, we also performed an intention-to-treat analysis (ITT) (Model 5). In the ITT analysis, participants were assigned to groups according to the cafeteria in which they completed the baseline questionnaire. Lastly, we performed a random split of the sample (Model 4). In conclusion, we provide 5 models:

- Model 1: unadjusted
- Model 2: adjusted for age, gender, relationship status, yearly income per household, educational degree, number of children in household, age of youngest child (years) and self-reported moderate intensity physical activity (h/week) [48, 53].
- Model 3: propensity score weighted model (propensity score based on the same covariates as in Model 2).
- Model 4: Random split.
- Model 5: Intention to treat analysis.

#### Intervention fidelity

Weekly frequency of cafeteria visits and information material uptake are presented in frequency tables. Vegan meal consumption is presented as a frequency table for the intervention group only, depending on the self-assigned dietary type at baseline.

#### Associations of the EAT-Lancet Planetary Health Diet Index with Well Being

Lastly, we performed an exploratory analysis of the relationship of the PHD-Index with the WEMWBS and SF-36 outcomes in the cross-sectional baseline sample (*N* = 563) (see Supplementary Fig. 3) using linear regression. We corrected for multiple testing, assigning the significance level to α = 0.008. If the outcomes showed a significant relationship, we performed multiple-adjusted linear regressions, including covariates with significant coefficients. We tested the significance of the R-square change of the previous model against the model with added coefficients using an F-test and only added coefficients under the significance level of α < 0.05. Further, we performed linear regressions for relationships of the change in the PHD-Index over the intervention period with the change in WEMWBS and SF-36 outcomes in the baseline and at follow-up sample (*N* = 182).

## Results

### Recruitment and data collection

Initially 838 participants were recruited for the study. Of those, 770 participants provided sufficient data to construct the WEMWBS score at baseline. For the quasi-experimental study, data from 249 participants were analysed (control group: *n* = 128, intervention group: *n* = 121). The drop-out rate was 68%. After removing outliers, we fell three participants short of reaching the sample size of 245.

### Baseline characteristics

The baseline characteristics for each group are presented in Table 1. There were no significant differences in age, gender identity, marital status, monthly income, number of children in the household, age of youngest child, and physical activity. However, the intervention group less frequently attained high educational degrees (61% vs. 64%) and more frequently consisted of students (10% vs. 2%). We identified moderate intensity physical activity (MIPA) as a potential confounder, because it is a time-varying covariable that cannot be adjusted for in this difference-in-differences analysis [48]. However, we see similar trends for MIPA in the intervention and in the control group. The percentages for the groups of participants with 2.5 to 5 h of MIPA increase by 3–5 percentage points for both groups. The distributions of self-assigned type of diet were similar between groups. In the intervention group at baseline, 4% followed a vegan diet, 13% adhered to a vegetarian diet, and 41% stated to eat flexitarian or pescetarian. The characteristics of the analytical sample did not differ significantly from those in the baseline sample. However, participants in the analytical sample were older, more likely to be married and had more children.

**Table 1:** Baseline characteristics of 190 participants, by intervention arm

Characteristics	Control n (%)	Intervention n (%)	p *
n	128	121	
**Age (years)**			0.10
18–29	20 (15.6)	26 (21.5)	
30–39	36 (28.1)	35 (28.9)	
40–49	34 (26.6)	16 (13.2)	
50–59	26 (20.3)	32 (26.5)	
60–79	12 (9.4)	12 (9.9)	
**Gender**			0.80
Male	45 (35.4)	41 (33.9)	
Female	82 (64.6)	80 (66.1)	
Diverse^a^	1	0	
**Relationship status**			0.65
Single or short-term relationship	45 (35.2)	49 (40.8)	
Single after long relationship	7 (5.5)	6 (5.0)	
In a long-term relationship	76 (59.4)	65 (54.2)	
Missing	0	1	
**Yearly income per household (€)**			0.99
0–19.999€	8 (6.3)	8 (6.6)	
20.000–59.999€	38 (29.7)	37 (30.6)	
60.000–99.999€	37 (28.9)	31 (25.6)	
100.000 € or more	26 (20.3)	26 (21.5)	
No answer	19 (14.8)	19 (15.7)	
Missing	0	0	
**Educational degree**			0.15
High: Higher education or higher vocational training	82 (64.1)	74 (61.2)	
Medium: A levels or completed vocational training	37 (28.9)	44 (36.4)	
Low: Secondary School/GCSE without vocational training	9 (7.0)	3 (2.5)	
Missing	0	0	
**Number of children in household**			0.33
No children	80 (62.5)	86 (71.1)	
1	20 (15.6)	16 (13.2)	
2 or more	28 (21.9)	19 (15.7)	
Missing	0	0	
**Age of youngest child (years)**			0.52
No children	61 (48.0)	64 (53.3)	
0 to 9	27 (21.3)	20 (16.7)	
10 to 17	17 (13.4)	10 (8.3)	
18 to 30	15 (11.8)	19 (15.8)	
Over 30	7 (5.5)	7 (5.8)	
Missing	1	1	
**Occupation**			0.01
Student/Voluntary year	3 (2.4)	12 (9.9)	
Employee or state official	124 (97.6)	109 (90.1)	
Missing	1	0	
**Moderate intensity physical activity (h/week) (self-reported)**			0.25
Less than 1 h/week	32 (25.0)	23 (19.0)	
1 to 2.5 h/week	46 (35.9)	56 (46.3)	
2.5 to 5 h/week	33 (25.8)	32 (26.5)	
Over 5 h/week	17 (13.3)	10 (8.3)	
Missing	0	0	
**Self-assigned Diet Type**^**b**^			0.87
Mixed Diet	53 (43.8)	48 (41.4)	
Flexitarian	47 (38.8)	43 (37.1)	
Pescetarian	6 (5.0)	5 (4.3)	
Vegetarian	12 (9.9)	15 (12.9)	
Vegan	3 (2.5)	5 (4.3)	
Missing	7	5	

Results are shown as number of participants (percentage)

^*^ *p*-values were calculated using Chi-Square-Tests

^a^ to maintain statistical validity of the Chi-Square Test and reducing the risk of bias, the person with diverse gender was included in the female gender category (mode) for the Chi-Square-Test

^b^ Mixed Diet = no restrictions. Flexitarian = meat consumption less than three times a week. Pescetarian = mixed diet excluding meat, including seafood. Vegetarian = mixed diet excluding meat and seafood. Vegan = diet excluding all products from animal sources (e.g., meat. seafood and dairy products)

### Feasibility and intervention fidelity

From those participants allocated to the intervention or the control cafeteria, 73% and 47% visited the cafeteria more than three times a week during the intervention period, respectively (Supplementary Table 2). Half of the participants in the intervention cafeteria (51%) consumed the new vegan menu one to two times per week, and 34% of the participants consumed this menu three to five times per week. Three-quarters of participants consuming mixed diets (74%) consumed the vegan menu at least once a week (Supplementary Table 3). However, the information material (flyer and recipe book) was only seen by 46% of the intervention group, and 21% of the control group stated that they had also seen the information material (Supplementary Table 4).

### Distributions of wellbeing outcomes

At baseline, the mean WEMWBS for the primary outcome mental wellbeing was 54.46 ± 7.13 score points in the control group and 55.19 ± 6.41 score points in the intervention group. For the secondary outcomes from the SF-36 pertaining to physical wellbeing and role limitations, we found no significant differences in mean values between groups except regarding RF. Here, the control group had a higher mean value than the intervention group. Table 2 shows means and standard deviations of the outcomes for all wellbeing outcomes.

**Table 2:** Distributions by intervention arm at baseline and at follow-up, within-group differences and intervention effects for Mental Wellbeing (WEMWBS), Physical Wellbeing and Role Limitations (SF36)

**Outcome**		**Intervention Group**				**Control group**				**Intervention effects**		
	**N**	**Baseline**	**Follow-up**	**WGD**^**a**^	**p**	**Baseline**	**Follow-up**	**WGD**^**a**^	**p**	**DID**^**b**^	**95% CI**	**p**
**Mental Wellbeing (WEMWBS)**												
Mental Wellbeing score	242	55.19 ± 6.41^**a**^	56.08 ± 6.40	0.89 ± 5.9	0.11	54.46 ± 7.13	54.75 ± 7.36	0.29 ± 5.04	0.52	0.61	-0.78, 1.99	0.39
**Physical Wellbeing (SF-36)**												
Global Self-rated Health	242	62.71 ± 19.27	63.35 ± 18.69	0.64 ± 17.13	0.69	62.1 ± 19.78	62.5 ± 19.52	0.4 ± 16.56	0.79	0.23	-3.56, 5.27	0.91
Physical functioning	239	92.95 ± 12.87	93.38 ± 12.28	0.43 ± 8.55	0.59	94.55 ± 14.26	93.85 ± 10.62	-0.7 ± 9.43	0.42	1.12	-1.17, 3.42	0.34
Bodily pain	243	83.49 ± 19.96	83.07 ± 22.14	-3.71 ± 18.93	0.03	86.73 ± 17.23	83.02 ± 19.93	-0.42 ± 16.28	0.78	3.29	-1.18, 7.76	0.15
**Role Limitations (SF-36)**												
Role-Physical ^c^	223	88.55 ± 23.61	89.02 ± 23.84	0.47 ± 18.49	0.79	94.83 ± 14.57	92.24 ± 19.06	-2.59 ± 15.94	0.08	3.05	-1.49, 7.60	0.19
Role-Emotional^d^	227	90.91 ± 20.15	90.00 ± 19.45	-0.91 ± 22.78	0.68	90.03 ± 22.43	89.46 ± 23.01	-0.57 ± 19.57	0.75	-0.34	-5.88, 5.20	0.90

Distribution outcomes are presented as means ± standard deviations. Within-group differences and their p-values were calculated by paired t-tests. DID estimates, their 95% confidence intervals (CIs) and p-values were calculated by linear regressions. For both analyses group (i.e. intervention vs control) was used as the exposure and the in-person pre-post differences in wellbeing measurements as the outcomes

^a^*WGD* within-group differences

^b^*DID *difference-in-differences estimate

^c^*Role-Physical *Role Limitations due to Physical Health

^d^*Role-Emotional *Role Limitations due to Emotional Problems

### Intervention effects on wellbeing outcomes

After the intervention, mean WEMWBS increased in the intervention group by 0.86 score points and increased in the control group by 0.29 score points, leading to a difference-in-differences of 0.61 (95% CI: − 0.78, 1.99; p = 0.39, Cohens d = 0.11). Table 2 shows the intervention effects from difference-in-differences analyses for all wellbeing outcomes. In the SF-36 outcomes, we saw no significant intervention effects.

### Sensitivity analyses

None of the dietary patterns had a significant effect on well-being (all p-values > 0.05). The week in which participants completed the endline assessment did not significantly influence their mental wellbeing scores. Furthermore, the distribution of endline assessment completion across weeks was similar in both groups. The fully adjusted Model 2 provided a slightly higher difference-in-differences estimate = 0.78.

(95% CI: − 0.67, 2.22; p = 0.29), as did the propensity-score-weighted difference-in-differences analysis (Model 3) (Table 3). All additional sensitivity analyses are presented in Table 3 and yielded similar and insignificant difference-in-differences estimates. Nearly all SF-36 outcomes showed similar and insignificant estimates from sensitivity analyses (see Supplementary Table 5 a-e).

**Table 3:** Distributions by intervention arm at baseline and at follow-up, within-group differences and intervention effects for Mental Wellbeing (WEMWBS) using five linear regression models: crude (1), adjusted (2), propensity score-weighted (3), random split (4) and intention-to-treat model (5)

**Model**		**Control Group**			**Intervention Group**			**Intervention effects**		
	**N**	**Baseline**	**Follow-up**	**WGD**^**a**^	**Baseline**	**Follow-up**	**WGD**^**a**^	**DID**^**b**^	**95% CI**	**p-value**
Model 1	242	54.46	54.75	0.29	55.19	56.08	0.89	**0.61**	-0.78, 1.99	0.39
Model 2	237	54.81	53.86	-0.95	55.64	55.47	-0.18	**0.78**	-0.67, 2.22	0.29
Model 3	242	54.53	54.64	0.11	55.17	56.05	0.88	**0.77**	-0.59, 2.13	0.27
Model 4	232	54.18	54.51	0.34	55.19	56.08	0.89	**0.56**	-0.87, 1.99	0.44
Model 5	242	54.64	54.81	0.16	55.01	56.08	1.07	**0.91**	-0.48, 2.30	0.20

^**a**^*WGD *within-group differences

^**b**^*DID *difference-in-differences estimate

Distribution outcomes are presented as means. Within-group differences were calculated by paired t-tests. DID estimates, their 95% confidence intervals (CIs) and p-values were calculated by linear regressions. For both analyses, group (i.e. intervention vs control) was used as the exposure and the in-person pre-post differences in mental wellbeing (WEMWBS)

### Association of the EAT-Lancet planetary health diet index with well being

Linear regression of the PHD-Index with mental wellbeing showed a relationship with a coefficient of 0.13 (95% CI: 0.03, 0.23; *p* = 0.015). (Table 4). We found no significant relationships with SF36-outcomes Bodily Pain, Physical functioning, Role-Emotion and Role-Physical. However, in the crude regression model, we saw a significant relationship of the PHD-Index with global self-rated health. When adjusting the regression with global self-rated health for age, moderate-intensity physical activity and gender, the relationship weakened from a coefficient of 0.48 (95% CI: 0.20, 0.75; *p* = < 0.001) to 0.43 (95% CI: 0.14, 0.71; *p* = 0.003). In the linear regressions of differences in wellbeing outcomes with the individual difference in PHD-Index between baseline and follow-up, we found no significant relationships (Supplementary Table 6).

**Table 4:** Linear Regression coefficients of the Planetary Health Diet Index (PHD-Index) with wellbeing outcomes at baseline among 563 participants

	**Crude Model (*N*= 563)**				**Adjusted Model (*N*= 545)**			
Outcome	R-Squared	**ß coefficient**	**95% CI**	**p-value**	R-Squared	**ß coefficient**	**95% CI**	**p-value**
**Mental Wellbeing (WEMWBS)**								
Mental Wellbeing	0.01	0.13	0.03, 0.23	0.015				
**Physical Wellbeing (SF-36)**								
Global Self-Rated Health^a^	0.02	0.48	0.20, 0.75	< 0.001	0.13	0.43	0.14, 0.71	0.003
Physical functioning	0.00	0.09	-0.07, 0.25	0.26				
Bodily pain	0.00	-0.22	-0.50, 0.07	0.13				
**Role Limitations (SF-36)**								
Role-Physical ^b^	0.00	-0.16	-0.53, 0.20	0.37				
Role-Emotional ^c^	0.00	0.2	-0.21, 0.62	0.34				

ß-coefficients, 95% confidence intervals (CIs) and p-values were calculated by linear regressions using the PHD-Index at baseline as the exposure and the wellbeing measurements at baseline as the outcomes. To account for multiple testing, we applied a corrected significance threshold of α = 0.008

^a^The adjusted model of the global self-rated health regression was adjusted for age, moderate-intensity physical activity and gender

^b^Role-Physical = Role Limitations due to Physical Health

^c^Role-Emotional = Role Limitations due to Emotional Problems

## Discussion

### Summary

The aim of this study was to determine the effects of a vegan food option on mental wellbeing and HRQoL among employees at Heidelberg University Hospital. Further, we established the relationships of the PHD-Index with these outcomes. While there were no significant intervention effects on mental wellbeing and HRQoL, higher adherence to the Planetary Health Diet was significantly associated with higher global self-rated health.

### Effects of the Sustainable Diet Intervention

#### Interpretation of our findings

The distribution of the types of diet in our sample was similar to the distribution in a representative German study in 2023, with slightly more individuals following vegan and vegetarian diets and slightly less on a flexitarian diet [9]. The mean WEMWBS in this study was 3–5 points higher than the mean scores in the health survey of England at baseline [54]. Also, the mean scores in the HRQoL outcomes were 5–10 points higher than the mean values for the US population [41]. In our intervention, the participants’ type of diet at baseline (mixed, flexitarian, pescatarian, vegetarian, vegan) did not influence the wellbeing trends.

While we observed only trends for sustainable diets to improve wellbeing, previous investigations have presented significant, beneficial associations. A study with a multi-component lifestyle intervention increasing physical activity and portions of fruits and vegetables per day found significant increases in median WEMWBS from 48 at baseline to 53 after 12 months.

[44]. Prospective studies implementing vegan diet interventions have shown significant increases in general health, physical functioning and mental wellbeing on the SF- 36 in their intervention groups compared with control groups, ranging from 5 to 10 points [42, 55]. Lastly, rheumatoid arthritis patients assigned to a diet excluding meat, gluten and lactose for three months showed a significant improvement in pain levels and overall states of physical and mental health (SF-36) [56]. In comparison to our study, these studies were characterized by either a longer intervention duration (1 year or longer), more fundamental dietary changes, or lower mental wellbeing levels. Future studies should be longer in duration when aiming to measure long-term effects of sustainable facility-based diet interventions and larger effects may be seen when limiting unsustainable food options.

### Strengths and limitations

The shift towards a more sustainable food environment constitutes an evolving real-life process. Thus, it has become particularly crucial to explore the effects of food environments on wellbeing. So far, previous studies on the effects of diet interventions on mental wellbeing or HRQoL have relied on comprehensive lifestyle interventions or individual counselling [42, 44, 55]. To our knowledge, the present study is the first which performed a facility-based diet intervention towards more sustainable diets, solely by changing the food environment. However, as one meat-and-fish menu a day was still offered in the intervention cafeteria, our intervention design was not as fundamental as other multi-component lifestyle interventions [42, 44, 55]. Some participants in the intervention group may not have undergone any dietary changes as non-vegan options were still available. In addition, the dietary intervention focused primarily on offering a vegan menu for lunch, while other meals in private life may have not changed. These could be reasons for the lack of effects in our study. Future studies on a facility-based diet intervention could therefore apply the Experience Sampling Method (ESM) [57], a digital diary-technique in order to monitor mood or food intake as well as the translation of the intervention to daily life.

Our sample size calculation was based on cross-sectional studies on the association of fruits and vegetables with the WEMWBS as well as an intervention study which implemented a multi-component lifestyle intervention to booster wellbeing including exercise and motivational coaching [15, 43–45]. The present intervention program was milder than those reported in the previous studies. This may have resulted in overestimating the possible effect of the intervention and underestimating the needed sample size. In this study, the difference-in-differences estimate for mental wellbeing was small and the resulting Cohens d in this study was very small. Therefore, we conclude that our sample size was too small and we encounter the probability of type II error with insufficient statistical power to detect an effect. Though a large proportion of individuals were lost to follow-up (68%) the characteristics between individuals lost to follow-up and those who remained in the study were similar; and there were similar characteristics and numbers between groups in the analysed sample. Therefore, selection bias appears to be unlikely. Further, we may have encountered ceiling effects, because all our outcomes had higher mean values than other population-based studies [34, 58].

We have controlled for potential confounders, observed and unobserved, by means of the difference-in-differences analysis. Yet, in contrast to randomized controlled trials, difference-in-differences analyses rely on the “parallel trends” assumption [50]. While we could not statistically investigate this assumption in our study, conceptual arguments, similar distributions of covariates and between-group distributions of the outcome variables at baseline favour this assumption. We excluded participants with changes above 70% in SF-36 wellbeing outcomes, which is unconventional. However, the outcomes remained insignificant in both datasets, with and without outliers. Hence, we opted to present mean differences less influenced by individual cases and closer to the population median differences of 0 [58]. Though the difference-in-differences method has been used previously to describe intervention effects on SF-36 outcomes, the known ceiling effects can make the interpretation of mean values difficult or misleading [47]. This has been discussed in detail by Velanovich [58] Therefore, for future studies on HRQoL, we recommend using the Quality of Wellbeing Scale (QWB), which has an approximately normal distribution for populations of adults [47].

Finally, spill-over effects due to individuals switching between cafeterias or sharing information among employees could have attenuated the actual effects of the intervention. We assume that most of the participants who switched between our groups, switched at random due to external factors or were individuals highly motivated to participate in the intervention. This could explain, why the effect estimates in the ITT analyses were stronger, though still insignificant. Also, the information component was not seen by most participants in the intervention group and was seen by some participants in the control group. Future studies who want to place a focus on the education component of a diet intervention should concentrate on a rigorous dissemination of information material. Additionally, though the week of participation in the endline assessment did not significantly influence our mental wellbeing outcome we propose future studies to perform endline assessment while the intervention is still running.

### Associations of the EAT-Lancet Planetary Health Diet

This study is also the first to investigate the relationship of a PHD-Index with mental wellbeing and HRQoL outcomes. Previous studies on the relationship of sustainable diet patterns with such outcomes commonly investigated diets high in fruits and vegetables or excluding meat and dairy products [42, 44, 55, 56] Different indices for the PHD concept, that have emerged since 2019, constitute promising tools for research on commonly defined sustainable diets. Of the currently available diet indices, the Stubbendorff index shows the best performance regarding the association with health risks and greenhouse gas emissions [59]. We reconstructed this index using self-reported dietary data from our FFQ. We acknowledge that FFQs can be subject to recall bias and underreporting and that our FFQ has not been validated. This highlights the importance of digital monitoring with high ecological validity and reduced recall bias. However, as all participants carry the same measurement error and can be ranked within the population, this should not affect our regression analyses.

In contrast to the lack of association of the PHD-Index with mental wellbeing, we saw a significant positive relationship with global-self-rated health. This adds to the growing body of knowledge on the association of the PHD with health [12, 60, 61]. Previous studies linked high fruit and vegetable consumption to improved mental wellbeing [44]. In a previous paper, we performed an analysis on a subsample of participants from this paper including an analysis of the intake of specific food components. There, we saw high mean levels of fruit and vegetable intake, but only modest PHD-Index scores (22/42 points) [32]. We conclude that participants with healthy but unsustainable diets may receive high wellbeing but low PHD-Index scores. Regarding mental wellbeing and Physical Functioning, Bodily Pain, Role-Emotional and Role-Physical, we conclude that the PHD neither over- nor underperforms when compared with other healthy diets. Further studies could take individual food categories of the PHD-Index, e.g. beef or legumes consumption, into account. Of course, causality and directionality cannot be established from cross-sectional analyses. However, the hypothesis that sustainable diets are better for mental wellbeing and HRQoL should be verified in longitudinal and experimental studies. Lu et al. have provided a starting step with their longitudinal research on the PHD-Index and mental health, showing that over more than ten years of follow-up the risk of incident depression, anxiety and their co-occurrence are lower in those with highest adherence to the Planetary Health Diet in comparison to those with lowest adherence.

### Recommendations for future research on sustainable diets and wellbeing

Neither the diet intervention nor the PHD-Index explained much variation in mental wellbeing and HRQoL models. Previous studies confirm that mental wellbeing is a complex construct influenced by many different factors and especially factors with a positive association have only a small influence on wellbeing [62]. Therefore, future studies could include more covariates, including health perception, loneliness [62], stress, mindfulness [63], and life events such as migration or bereavement [64]. Furthermore, there is mixed evidence as to whether people generally following sustainable diets show more positive or negative mental health [65]. This could be attributed to factors influencing mental health that are specifically prevalent in people following sustainable diets. Vegetarians and vegans are often young and female [65]. Also, they are often more attuned to ethical and environmental issues [65]. Individuals who deeply engage with ethical and environmental concerns may experience greater psychological distress compared to those less focused on such issues [66] and following these diets can lead to social stigmatization [67]. Lastly, work productivity, mental well-being and HRQoL can also be mediated by the weight loss and improved health markers which are often associated with the adaptation of such diets [68, 69]. Thus, future research should also include covariates like worldviews, stigmatization and physical health improvements. To attain more generalizable findings, intervention studies should include multiple intervention sites or examine a more diverse population.

## Conclusion

Promoting sustainable diets in the workplace is necessary to maintain the health of our environment. Still, our study could not prove the same beneficial effects for mental wellbeing and HRQoL among consumers of sustainable diets. At the same time, our study showed a significant beneficial relationship between the PHD-Index and global self-rated health. Multiple other studies have shown that diets similar to the PHD provide benefits for mental wellbeing and HRQoL. Because a change in human diets, especially in countries with high meat-consumption, becomes increasingly important for planetary health, it is of utmost relevance to put dietary interventions with lasting effects and a successful translation into daily life into practice.

## Supplementary Information

Additional file 1.

## Data Availability

Anonymized participants’ data described in the manuscript will be made available upon reasonable request from the principal investigators and for research purposes only. An analysis proposal will be reviewed and examined regarding its compliance with the scientific goals of the KliOL project by the principal investigator.

## Abbreviations

DID: Difference-in-difference
HRQoL: Health-related Quality of Life
MIPA: Moderate Intensity Physical Activity
PHD: EAT-Lancet Planetary Health Diet
PHD-Index: EAT-Lancet Planetary Health Diet Index
SF-36: Short Form 36 Questionnaire
WEMWBS: Warwick Edinburgh Mental Well-being Scale
