# Supplementary material for: Effects of a cafeteria-based sustainable diet intervention on wellbeing at a large German hospital: a quasi-experimental study
Source: BMC Public Health. 2025 Jun 2;25:2047. doi: 10.1186/s12889-025-22533-6 (PMC12128345; doi:10.1186/s12889-025-22533-6)
Supplement: Supplementary file 1 — Additional file 1. [file 12889_2025_22533_MOESM1_ESM.docx]

**Supplement to**

# Effects of a cafeteria-based Sustainable Diet Intervention on wellbeing at a large German hospital: A Quasi-Experimental Study

Laura Harrison, Alina Herrmann, Claudia Quitmann, Gabriele Stieglbauer, Christin Zeitz, Ulrich Reininghaus, Anita Schick, Ina Danquah

# Table of Contents

[Effects of a cafeteria-based Sustainable Diet Intervention on wellbeing at a large German hospital: A Quasi-Experimental Study 1](#_Toc190098094)

[Table of Contents 2](#_Toc190098095)

[Supplementary Figure 1 Menu for one week of the intervention 3](#_Toc190098096)

[Supplementary Figure 2 Information flyer on sustainable nutrition and health 4](#_Toc190098097)

[Supplementary Table 1 Calculation of food groups for the EAT-Lancet Planetary Health Diet Index by Stubbendorff et al.1 (Target intake 2, calculation and scoring). 5](#_Toc190098098)

[Supplementary Table 2 Frequency of consumption of meals at the Control Cafeterias or Intervention Cafeteria (N=249) 6](#_Toc190098099)

[Supplementary Table 3 Frequency of Vegan Meal Consumption (missing=1)in the Intervention Cafeteria, by self-reported type of diet at baseline (missing=5) (N=115) 6](#_Toc190098100)

[Supplementary Table 4 Uptake of Information Material in Control and Intervention Group (N=226, missing=23) 6](#_Toc190098101)

[Supplementary Table 5 Distributions by intervention arm at baseline and at follow-up, within-group differences and intervention effects for SF-36 wellbeing outcomes using five linear regression models: crude (1), adjusted (2), propensity score-weighted (3), random split (4) and intention-to-treat model (5) 7](#_Toc190098102)

[Supplementary Figure 3 Flow diagram of excluded participants for the associations of PHD-Index and change in PHD-Index during the intervention period with wellbeing outcomes 9](#_Toc190098103)

[Supplementary Table 6 Linear regression coefficents of the in-person pre-post-difference in PHD-Index with the in-person pre-post-difference in wellbeing outcomes among 182 participants 10](#_Toc190098104)

[Bibliography 11](#_Toc190098105)

# Supplementary Figure 1 Menu for one week of the intervention

# Supplementary Figure 2 Information flyer on sustainable nutrition and health


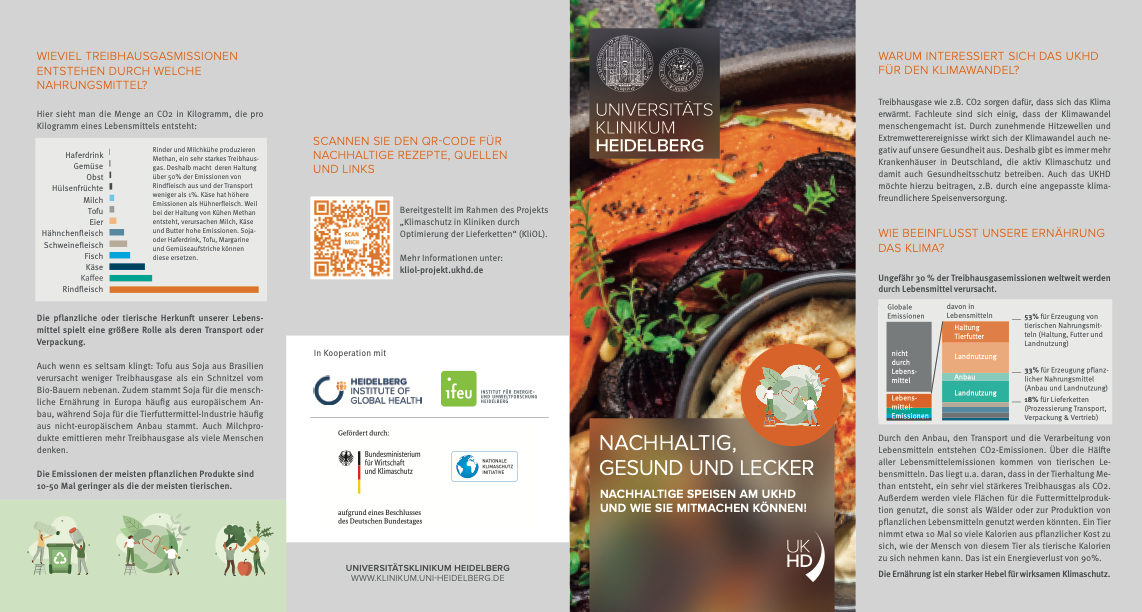


# Supplementary Table 1 Calculation of food groups for the EAT-Lancet Planetary Health Diet Index by Stubbendorff et al.1 (Target intake 2, calculation and scoring).

| **Food group & *Target intake (g)**** | **Calculation of food group** | **3 points** | **2 points** | **1 point** | **0 points** |
| --- | --- | --- | --- | --- | --- |
| **Limited intake** | | | | | |
| Beef and lamb  *7 (0–14)* | Beef, lamb, minced meat with pork and lamb, processed meats with beef and lamb including sausages. | <7 | 7–14 | 14–28 | >28 |
| Pork  *7 (0–14)* | Pork, minced meat of pork, processed meats with pork including ham, bacon, and sausages. | <7 | 7–14 | 14–28 | >28 |
| Chicken  *29 (0–58)* | Chicken and other poultry. | <29 | 29–58 | 58–116 | >116 |
| Eggs  *13 (0–25)* | Eggs e.g. boiled, fried. We included egg from recipes (e.g. cakes) and multiplied it by the factor derived from a question on the frequency of consumption of egg in sweet foods. | <13 | 13–25 | 25–50 | >50 |
| Dairy  *250 (0–500)* | Whole milk or derivative equivalents. Regular milk, low-fat milk, yoghurt and other fermented milk products, hard cheese, soft cheese, cream, butter, butter-based spreads. In the EAT-Lancet diet all dairy foods are expressed as of milk equivalents. The milk equivalents we used are based on the approach used by Stockholm Resilience Centre, based on ‘total solids’ and intakes of different dairy products were consequently multiplied with the following factors; whole milk 1.0, Cheese 5.0, cream 2.7 and butter 6.5. Furthermore, we included dairy from recipes (e.g., cakes) and multiplied it by the factor derived from a question on the frequency of consumption of dairy in sweet foods. | <250 | 250–500 | 500–1000 | >1000 |
| Potatoes  *50 (0–100)* | Boiled potatoes, fried potatoes, potatoes included in dishes such as potato salad. | <50 | 50–100 | 100–200 | >200 |
| Added sugar  *31 (0–31)* | Calculation of added sugar was performed by summing the amount of added sugars in all food items. Information on added sugars was obtained from openfoodfacts.org ^3^ using the nutritional information of a random exemplary product for each food item or using exemplary recipes for food items like cakes or cookies. | <31 | 31–62 | 62–124 | >124 |
| **Emphasised intake** | | | | | |
| Vegetables  *300*  *(200-600)* | All vegetables except legumes | >300 | 200-300 | 100-200 | <100 |
| Fruits  *200*  *(100–300)* | Fruits and berries | >200 | 100–200 | 50–100 | <50 |
| Whole grains  232 | Fiber-rich breakfast cereals (≥10% fiber), rolled oats, fiber-rich soft bread (>4.5% fiber), fiber-rich crispbread (≥10% fiber), fiber-rich rusks >10% fiber. We multiplied the intake for rice and pasta with a factor for the individual percentage of whole grain pasta and rice, derived from a question on the frequency of consumption of whole grain pasta and rice. | >232 | 116–232 | 58–116 | <58 |
| Fish  *28 (0–100)* | Fatty fish, lean fish, fish products, shellfish | >28 | 14–28 | 7–14 | <7 |
| Legumes  *75 (0–150)* | Dry beans, lentils, peas, soy. Targets and index refer to raw weight. Peas, lentils, beans, tofu, soy containing meat replacement products. | >75 | 37.5–75 | 18.75–37.5 | <18.75 |
| Nuts  *50 (0–100)* | Peanuts or tree nuts. All nuts and seeds including peanuts, nut mixes such as nut paste and chocolate cream. | >50 | 25–50 | 12.5–25 | <12.5 |
| Unsaturated oils  *40 (20–80)* | All plant oils and plant margarines. We added 28g (2 tablespoons) of fat to each diet. This was multiplied by a factor for the percentage of unsaturated fats, derived from a question on choices of oils and fats. | >40 | 20–40 | 10–20 | <10 |
| All plant drinks were included in respective categories (whole grains, fruits, nuts, legumes) and multiplied by 0.1 as they consist of 10% plant and 90% water. Plant-based alternatives were included in respective categories depending on the main plant-basis. | | | | | |

* The target intake for the EAT-Planetary Health Diet gives a range of possible macronutrient intakes per group to ensure healthy nutrition while staying within planetary boundaries. The suitable amount of intake for one food group may differ depending on the intake levels for other food groups, e.g., 58g/day chicken intake could be suitable if there is no cheese intake.

# Supplementary Table 2 Frequency of consumption of meals at the Control Cafeterias or Intervention Cafeteria (N=249)

| **Frequency of consumption of cafeteria meals**  **in number of individuals**  (**percentage per group)** | **Control Group (n=128)** |  | **Intervention Group (n=121)** | |
| --- | --- | --- | --- | --- |
|  | **Baseline** | **Follow-Up** | **Baseline** | **Follow-Up** |
| **Never or less than once a week** | 13 (10%) | 0 (0%) | 9 (7%) | 0 (0%) |
| **Once or twice a week** | 50 (39%) | 68 (53% ) | 34 (28%) | 33 (27%) |
| **More than three times a week** | 65 (51%) | 60 (47%) | 78 (64%) | 88 (73%) |

#

# Supplementary Table 3 Frequency of Vegan Meal Consumption (missing=1)in the Intervention Cafeteria, by self-reported type of diet at baseline (missing=5) (N=115)

| **Vegan Menu Consumption**  **in number of individuals**  (**percentage per group)** | **Type of diet stated at baseline** | | | | | |
| --- | --- | --- | --- | --- | --- | --- |
|  | **Mixed Diet**  **(n=47)** | **Flexitarian**  **(n=43)** | **Pescetarian (n=5)** | **Vegetarian (n=15)** | **Vegan (n=5)** | **Total**  **(N=115)** |
| **Never** | 12 (26%) | 3 (7%) | 1 (20% ) | 1 (7%) | 0 (0%) | **17 (15%)** |
| **Once or twice a week** | 27 (57%) | 23 (53% ) | 2 (40%) | 6 (40%) | 1 (20%) | **59 (51%)** |
| **Three to five times a week** | 8 (17%) | 17 (40% ) | 2 (40%) | 8 (53%) | 4 (20%) | **39(34%)** |

# Supplementary Table 4 Uptake of Information Material in Control and Intervention Group (missing=23, N=226)

| **Participants who...**  **in number of individuals**  (**percentage per group)** | **Control Group (n=114)** | **Intervention Group (n=112)** |  |
| --- | --- | --- | --- |
| **read the flyer** | 11(10%) | 27 (24%) |  |
| **read the recipe book** | 0 (0%) | 12 (11%) |  |
| **saw but did not read the flyer/recipe book** | 13 (11%) | 22 (20%) |  |
| **did not see the flyer/recipe book** | 90 (79%) | 60 (54%) |  |

Multiple choices were possible. Percentages (%) were calculated as all those that stated “yes” out of the participants per group.

# Supplementary Table 5 Distributions by intervention arm at baseline and at follow-up, within-group differences and intervention effects for SF-36 wellbeing outcomes using five linear regression models: crude (1), adjusted (2), propensity score-weighted (3), random split (4) and intention-to-treat model (5)

| **a.      Global Self-Rated Health** | | | | | | | | | | |
| --- | --- | --- | --- | --- | --- | --- | --- | --- | --- | --- |
| **Model** |  | **Control Group** | | | **Intervention Group** | | | **Intervention effects** | | |
|  | **N** | **Baseline** | **Follow-up** | **WGD^a^** | **Baseline** | **Follow-up** | **WGD^a^** | **DID ^b^** | **95% CI** | **p-value** |
| Model 1 | 242 | 62.10 | 62.50 | 0.40 | 62.71 | 63.35 | 0.64 | **0.23** | -4.03, 4.50 | 0.91 |
| Model 2 | 237 | 67.17 | 58.42 | -8.75 | 67.64 | 59.76 | -7.88 | **0.87** | -3.77, 5.51 | 0.71 |
| Model 3 | 242 | 61.98 | 62.44 | 0.46 | 62.42 | 63.41 | 0.99 | **0.53** | -3.67, 4.74 | 0.8 |
| Model 4 | 227 | 61.95 | 62.17 | 0.22 | 62.72 | 64.25 | 1.54 | **1.31** | -3.04, 5.66 | 0.55 |
| Model 5 | 242 | 63.55 | 63.55 | 0.00 | 61.04 | 62.16 | 1.13 | **1.13** | -3.15, 5.40 | 0.6 |
| **b.     Physical functioning** | | | | | | | | | | |
| **Model** |  | **Control Group** | | | **Intervention Group** | | | **Intervention effects** | | |
|  | **N** | **Baseline** | **Follow-up** | **WGD^a^** | **Baseline** | **Follow-up** | **WGD^a^** | **DID ^b^** | **95% CI** | **p-value** |
| Model 1 | 239 | 94.55 | 93.85 | -0.70 | 92.95 | 93.38 | 0.43 | **1.12** | -1.17, 3.42 | 0.34 |
| Model 2 | 234 | 92.40 | 87.16 | -5.24 | 90.82 | 87.22 | -3.60 | **1.64** | -0.75, 4.03 | 0.18 |
| Model 3 | 239 | 94.61 | 93.71 | -0.90 | 92.84 | 93.42 | 0.58 | **1.49** | -0.93, 3.90 | 0.23 |
| Model 4 | 225 | 94.59 | 94.14 | -0.45 | 92.94 | 93.77 | 0.83 | **1.28** | -0.96, 3.53 | 0.26 |
| Model 5 | 239 | 94.85 | 94.23 | -0.62 | 92.48 | 92.89 | 0.41 | **1.03** | -1.28, 3.33 | 0.38 |
| **c.      Bodily Pain** | | | | | | | | | | |
| **Model** |  | **Control Group** | | | **Intervention Group** | | | **Intervention effects** | | |
|  | **N** | **Baseline** | **Follow-up** | **WGD^a^** | **Baseline** | **Follow-up** | **WGD^a^** | **DID ^b^** | **95% CI** | **p-value** |
| Model 1 | 243 | 86.73 | 83.02 | -3.71 | 83.49 | 83.07 | -0.42 | **3.29** | -1.18, 7.76 | 0.15 |
| Model 2 | 238 | 81.6 | 76.42 | -5.18 | 78.07 | 76.66 | -1.41 | **3.77** | -1.01, 8.54 | 0.12 |
| Model 3 | 243 | 86.97 | 82.61 | -4.37 | 83.70 | 82.53 | -1.17 | **3.20** | -1.20, 7.60 | 0.15 |
| Model 4 | 228 | 86.84 | 82.88 | -3.96 | 84.02 | 84.26 | 0.24 | **4.20** | -0.31, 8.71 | 0.07 |
| Model 5 | 243 | 87.29 | 83.21 | -4.08 | 82.63 | 82.86 | 0.22 | **4.31** | -0.16, 8.78 | 0.06 |
| **d.     Role Limitations due to Physical Health** | | | | | | | | | | |
| **Model** |  | **Control Group** | | | **Intervention Group** | | | **Intervention effects** | | |
|  | **N** | **Baseline** | **Follow-up** | **WGD^a^** | **Baseline** | **Follow-up** | **WGD^a^** | **DID ^b^** | **95% CI** | **p-value** |
| Model 1 | 223 | 94.83 | 92.24 | -2.59 | 88.55 | 89.02 | 0.47 | **3.05** | -1.49, 7.60 | 0.19 |
| Model 2 | 218 | 86.64 | 80.28 | -6.36 | 79.66 | 76.87 | -2.79 | **3.57** | -1.09, 8.23 | 0.13 |
| Model 3 | 223 | 94.76 | 92.25 | -2.52 | 88.13 | 89.23 | 1.11 | **3.62** | -0.96, 8.20 | 0.12 |
| Model 4 | 210 | 94.52 | 91.43 | -3.10 | 89.05 | 89.76 | 0.71 | **3.81** | -0.96, 8.58 | 0.12 |
| Model 5 | 223 | 94.76 | 92.34 | -2.42 | 88.13 | 88.64 | 0.51 | **2.92** | -1.65, 7.50 | 0.21 |

Table continues on page 7

| **e.      Role Limitations due to Emotional Problems** | | | | | | | | | | |
| --- | --- | --- | --- | --- | --- | --- | --- | --- | --- | --- |
| **Model** |  | **Control Group** | | | **Intervention Group** | | | **Intervention effects** | | |
|  | **N** | **Baseline** | **Follow-up** | **WGD^a^** | **Baseline** | **Follow-up** | **WGD^a^** | **DID ^b^** | **95% CI** | **p-value** |
| Model 1 | 227 | 90.03 | 89.46 | -0.57 | 90.91 | 90.00 | -0.91 | **-0.34** | -5.88, 5.2 | 0.90 |
| Model 2 | 222 | 92.85 | 91.11 | -1.74 | 94.05 | 92.38 | -1.67 | **0.07** | -5.79, 5.94 | 0.98 |
| Model 3 | 227 | 90.00 | 89.11 | -0.88 | 91.02 | 89.83 | -1.19 | **-0.31** | -5.85, 5.23 | 0.91 |
| Model 4 | 213 | 88.99 | 88.99 | 0.00 | 91.28 | 90.34 | -0.93 | **-0.93** | -6.51, 4.64 | 0.74 |
| Model 5 | 227 | 90.32 | 88.17 | -2.15 | 90.61 | 91.59 | 0.97 | **3.12** | -2.43, 8.67 | 0.27 |

**^a^** WGD= within-group differences

**^b^** DID=difference-in-differences estimate

Distribution outcomes are presented as means. Within-group differences were calculated by paired t-tests. DID estimates, their 95% confidence intervals (CIs) and p-values were calculated by linear regressions. For both analyses, group (i.e. intervention vs control) was used as the exposure and the in-person pre-post differences in wellbeing measurements (SF-36) as the outcomes.

Legend:
Model 1: Unadjusted Model
Model 2: Adjusted for age, gender, relationship status, yearly income per household, educational degree, number of children in household, age of youngest child (years) and self-reported moderate intensity physical activity (h/week), “diverse” coded as missing
Model 3: Propensity-Score-Weighted (includes all covariates in Model 2, missing covariates and “diverse” were imputed with the sample mode)
Model 4: Random Split
Model 5: Intention-to-Treat-Analysis

# Supplementary Figure 3 Flow diagram of excluded participants for the associations of PHD-Index and change in PHD-Index during the intervention period with wellbeing outcomes

# Supplementary Table 6 Linear regression coefficients of the in-person pre-post-difference in PHD-Index with the in-person pre-post-difference in wellbeing outcomes among 182 participants

|  | **Crude Model** | |  |  | |
| --- | --- | --- | --- | --- | --- |
| Outcome (N=182) | R-squared | **ß coefficient** | **95% CI** | | **p-value** |
| WEMWBS | 0.00 | -0.01 | -0.27, 0.26 | 0.97 | |
| **Physical Well-being** |  |  |  |  | |
| Global Self-Rated Health | 0.00 | 0.22 | -0.51, 0.94 | 0.55 | |
| Physical functioning | 0.02 | 0.40 | -0.03, 0.83 | 0.07 | |
| Bodily pain | 0.00 | -0.20 | -1.00, 0.60 | 0.63 | |
| **Role Limitations** |  |  |  |  | |
| Role-Physical ^a^ | 0.01 | 0.64 | -0.61, 1.89 | 0.32 | |
| Role-Emotional ^b^ | 0.00 | 0.05 | -1.16, 1.27 | 0.93 | |

^a^ Role-Physical= Role Limitations due to Physical Health

^b^ Role-Emotional= Role Limitations due to Emotional Problems

ß-coefficients, 95% confidence intervals (CIs) and p-values were calculated by linear regressions using the pre-post difference in PHD-Index as the exposure and the pre-post differences in wellbeing measurements as the outcomes. To account for multiple testing, we applied a corrected significance threshold of α = 0.008.

# Bibliography

1. Stubbendorff A, Sonestedt E, Ramne S, Drake I, Hallström E, Ericson U. Development of an EAT-Lancet index and its relation to mortality in a Swedish population. *The American Journal of Clinical Nutrition* 2022; **115**(3): 705-16.

2. Willett W, Rockstrom J, Loken B, al e. Food in the Anthropocene: the EAT-Lancet Commission on healthy diets from sustainable food systems. *Lancet* 2019; **393**(10170): 447-92.

3. Open Food Facts - Freie Datenbank für Lebensmittel. <https://de.openfoodfacts.org/>.
